# The increase of NADH fluorescence lifetime is associated with the metabolic change during osteogenic differentiation of human mesenchymal stem cells (hMSCs)

**DOI:** 10.1186/1753-6561-6-S3-P51

**Published:** 2012-06-01

**Authors:** Han Wen Guo, Jia Sin Yu, Shu Han Hsu, Yau Huei Wei, Oscar K Lee, Hsing Wen Wang

**Affiliations:** 1 Institute of Biophotonics, National Yang Ming University, Taipei 112, Taiwan; 2 Department of Biochemistry and Molecular Biology, National Yang-Ming University, Taipei 112, Taiwan; 3 Department of Medicine, Mackay Medical College, San-Jhih, Taipei County 252, Taiwan; 4 Institute of Clinical Medicine, National Yang Ming University, Taipei 112, Taiwan

## Background

Fluorescence lifetime of NADH had been proposed to use as an intrinsic biomarker for monitoring cellular metabolism. In our pervious studies, we have demonstrated that NADH lifetime of hMSCs increase gradually with time of osteogenic differentiation. In this study, we performed NADH lifetime measurement of hMSCs from a different donor and studied the association with several metabolic indices such as ATP level, oxygen consumption and lactate release. We also measured the quantity of Complex I, III, IV and V during hMSC differentiation.

## Materials and methods

NADH fluorescence lifetime images were performed as our previous studies [1]. In brief, treated hMSC cells were imaged with a two-photon laser scanning microscope and with a 60 × 1.45 NA PlanApochromat oil objective lens (Olympus Corp., Japan). NADH fluorescence was excited at 740 nm by a Verdi pumped modelocked femtosecond Ti:sapphire laser (Coherent, Inc., Santa Clara, California) at 76 MHz and the emitted fluorescent light was detected at 450±40 nm by a bandpass filter (Edmund Optics, Inc., Barrington, New Jersey). Fluorescence photons were detected by a photon-counting photomultiplier H7422P-40 (Hamamatsu Photonics K.K., Hamamatsu, Japan). Time-resolved detection was conducted by the single-photoncounting SPC-830 printed circuit board (Becker & Hickl GmbH, Berlin, Germany). Data were analyzed with the commercially available SPCImage v2.8 software (Becker & Hickl GmbH, Berlin, Germany) via a convolution of the two component exponential decay function and the instrument response function (IRF), and then the convolved result were fitted to the actual data to derive lifetime parameters τ1 (NADH short lifetime component), τ2 (NADH long lifetime component), a1 (amplitude related to τ1), a2 (amplitude related to τ2), and τm. Mean lifetime τm is defined as (a1τ1+a2τ2)/(a1+a2). IRF was measured using a second-harmonic generated signal from a periodically poled lithium niobate crystal. The cell respiration rate was measured by a 782 Oxygen Meter as previously reported [2] and intercellular ATP level was measured by the bioluminescent somatic cell ATP assay kit (Sigma-Aldrich, St. Louis, Missouri).

## Results

The results show that during differentiation more oxygen consumption, higher ATP level expressed and less lactate released. Similar to our previous study, NADH fluorescence lifetime increased gradually during osteogenic differentiation and until 4 weeks after differentiation The increase of NADH lifetime was associated with ATP level and oxygen consumption (R^2^= 0.88 and 0.95 respectively). Significant higher expression of the total Complex protein than controls was observed at 3 and 4 weeks after differentiation. However, Complex I expression, which was believed to directly related to NADH, did not show significant correlation with the increase of NADH fluorescence lifetime. In summary, we demonstrated that the change of NADH lifetime was associated with the metabolic change during osteogenic differentiation of hMSCs. The increase of NADH lifetime was in part due to the increased Complex protein interaction in mitochondria during differentiation.
